# Comprehensive effects of aerobic training and dietary intervention on anthropometric and body composition parameters in obese adolescents

**DOI:** 10.1097/MD.0000000000047320

**Published:** 2026-01-23

**Authors:** Yahui Chang, Bo Zhou, Yalan Zhang, Ziyang He, Haoxuan Song, Yang Wang, Shurui Li, Meng Su

**Affiliations:** a School of Physical Education, Shanxi University, Taiyuan, Shanxi Province, China; b Physical Examination Center, Shanxi Coal Central Hospital, Taiyuan, Shanxi Province, China; c School of Physical Education and Sports Science, Nanjing Normal University, Nanjing, Jiangsu Province, China; d Key Laboratory of Ministry of Education of Exercise and Physical Fitness, Beijing Sport University, Beijing, China; e Shanxi Pharmaceutical Vocational College, Taiyuan, Shanxi Province, China.

**Keywords:** adolescent obesity, aerobic training, body composition, body mass index, nutritional intervention, physical fitness

## Abstract

Adolescent obesity has become a growing global health concern, contributing to the early development of metabolic and cardiovascular diseases. This study aimed to evaluate the comprehensive effects of aerobic training combined with dietary intervention on anthropometric indices, body composition, and functional outcomes in obese adolescents. This retrospective study analyzed adolescents with obesity who received treatment between January 2021 and December 2024. A total of 129 adolescents with obesity who received treatment between January 2021 and December 2024 were retrospectively analyzed. Participants were assigned to a control group (aerobic exercise only, n = 61) or an observation group (aerobic exercise combined with nutritional intervention, n = 68). Both groups underwent a 12-week structured exercise program, while the observation group additionally received individualized nutritional guidance tailored to caloric needs and macronutrient balance. Anthropometric parameters, body composition, cardiopulmonary function, and physical fitness were assessed before and after the intervention. After 12 weeks, the observation group demonstrated significantly greater reductions in body mass index, waist circumference, body fat percentage, and visceral fat area compared with the control group (all *P* < .01). In addition, lean body mass, step test performance, vital capacity, and multiple measures of physical fitness, including muscular endurance, flexibility, and agility, showed more pronounced improvement in the observation group. These findings indicate that the combination of aerobic exercise and dietary intervention produces synergistic benefits, resulting in more effective reductions in adiposity and enhanced cardiopulmonary and physical performance. However, the retrospective and non-randomized design may limit the interpretation of causality. Integrating structured nutritional management with exercise training may represent an optimal, non-pharmacological strategy for the prevention and treatment of adolescent obesity.

## 1. Introduction

Adolescent obesity has emerged as a major global public health concern, with its prevalence increasing dramatically over the past 3 decades. According to the World Health Organization the prevalence of overweight and obesity among adolescents aged 10 to 19 years has more than quadrupled since 1975, with an estimated 340 million adolescents affected worldwide by 2022. Obesity during adolescence is associated not only with increased cardiometabolic morbidity but also with psychosocial consequences that extend into adulthood, including elevated risks of hypertension, type 2 diabetes mellitus, dyslipidemia, nonalcoholic fatty liver disease, and early atherosclerotic changes.^[1–3]^ Furthermore, obese adolescents often exhibit reduced exercise capacity, muscle performance, and pulmonary function, which contribute to diminished physical fitness and overall quality of life. Thus, early and effective intervention during adolescence is critical for interrupting the trajectory toward adult obesity and its related complications. Lifestyle modification remains the cornerstone of obesity management in pediatric and adolescent populations. Among lifestyle interventions, aerobic exercise and dietary modification are 2 of the most evidence-based strategies for reducing adiposity and improving metabolic health. Aerobic training enhances cardiorespiratory fitness, increases energy expenditure, and facilitates lipid oxidation, thereby contributing to reductions in total and visceral adipose tissue. Nutritional interventions, particularly those focusing on caloric restriction and macronutrient optimization, play a pivotal role in regulating energy balance, improving insulin sensitivity, and maintaining lean body mass during weight reduction.^[4,5]^ However, despite the established benefits of both modalities, their combined application in adolescent populations has been less systematically evaluated. Most existing studies have examined either exercise or diet in isolation, and data directly comparing their individual and synergistic effects on anthropometric and functional outcomes remain limited.

Recent evidence suggests that multimodal interventions integrating structured aerobic exercise with nutritional counseling yield superior outcomes compared with single-component strategies. Controlled trials in pediatric cohorts have demonstrated that combined lifestyle interventions produce greater reductions in body mass index (BMI) and waist circumference, larger decreases in visceral adipose tissue, and more pronounced improvements in cardiometabolic biomarkers. Furthermore, such interventions have been associated with enhanced muscle strength, flexibility, and endurance – factors essential for promoting sustained physical activity and long-term weight maintenance. Nevertheless, the magnitude and pattern of improvement in physical function and body composition parameters in obese adolescents receiving combined versus isolated interventions are not yet fully characterized, particularly in Asian populations where body fat distribution and metabolic responses may differ from Western cohorts.^[6–8]^ The physiological mechanisms underlying the combined benefits of aerobic exercise and nutritional regulation are multifactorial. Aerobic exercise promotes mitochondrial biogenesis, augments oxidative enzyme activity, and increases skeletal muscle insulin sensitivity, while dietary control reduces lipogenesis and chronic low-grade inflammation. Together, these adaptations enhance substrate metabolism and improve body composition by reducing adipose tissue mass and preserving lean body mass. Moreover, improved cardiopulmonary efficiency resulting from regular aerobic exercise may facilitate greater exercise tolerance, enabling adolescents to achieve higher training volumes and energy deficits, thereby potentiating the overall therapeutic effect.^[9,10]^

Given these considerations, the present study aimed to comprehensively evaluate the effects of a 12-week program of aerobic exercise combined with nutritional intervention on anthropometric indices, body composition, cardiopulmonary function, and physical fitness in adolescents with obesity. This study provides novel evidence from an Asian adolescent population, where body fat distribution and metabolic responses may differ from Western cohorts. Moreover, few studies have simultaneously evaluated anthropometric indices, visceral fat area, cardiopulmonary function, and physical fitness within a single analytical framework. These aspects represent key innovations of the present research.

## 2. Methods

### 2.1. Study design

This study was approved by the Ethics Committee of Shanxi Pharmaceutical Vocational College. The present study was designed as a retrospective clinical analysis. A total of adolescent patients diagnosed with obesity who received treatment at our hospital between January 2021 and December 2024 were included. Patients treated between January 2021 and December 2022, who underwent aerobic exercise guidance alone, were assigned to the control group, whereas those treated between January 2023 and December 2024, who received combined aerobic exercise guidance and nutritional intervention, were assigned to the observation group. Inclusion criteria were as follows: patients aged 12 to 18 years with a diagnosis of obesity according to the age- and sex-specific BMI cutoff values recommended by the World Health Organization; availability of complete clinical, anthropometric, and laboratory data; and adherence to the prescribed intervention program for at least 12 consecutive weeks. Exclusion criteria included: secondary obesity due to endocrine, genetic, or medication-related causes (e.g., hypothyroidism, Cushing syndrome, or corticosteroid use); presence of severe cardiovascular, hepatic, renal, or psychiatric disorders; incomplete clinical data or loss to follow-up; and refusal or inability to participate in the intervention program. The study was conducted in accordance with the ethical principles outlined in the Declaration of Helsinki and was approved by the Institutional Medical Ethics Committee of our hospital. Written informed consent was obtained from all participants and their legal guardians prior to inclusion. This is a retrospective analysis based on all eligible cases during the research period. However, we conducted a sample size calculation, and the sample size included in this study is sufficient.

### 2.2. Intervention protocol

All participants received standardized guidance under professional supervision throughout the study period. Interventions were conducted over a 12-week period for both groups.

Control group (aerobic exercise intervention): Patients in the control group received structured aerobic exercise training under the supervision of certified exercise physiologists. The aerobic training program consisted of moderate-intensity exercises such as brisk walking, jogging, or cycling, performed 5 times per week, 40 to 60 minutes per session. Exercise intensity was maintained at 60% to 75% of the individual’s maximum heart rate, monitored using wearable heart rate devices. Warm-up and cool-down sessions of 5 to 10 minutes were included before and after each exercise session to prevent musculoskeletal injury. Participants were instructed to maintain their usual dietary habits during the intervention period. Compliance was monitored weekly through attendance records and self-reported exercise logs.

Observation group (aerobic exercise + nutritional intervention): In addition to the same aerobic exercise regimen as the control group, patients in the observation group received individualized nutritional intervention designed and monitored by registered dietitians. The dietary plan was developed according to the patient’s age, sex, BMI, basal metabolic rate, and daily physical activity level. Total daily caloric intake was reduced by approximately 20% to 25% from estimated energy requirements, with macronutrient distribution set at 50% to 55% carbohydrates, 25% to 30% fats, and 15% to 20% proteins. Participants were advised to increase intake of high-fiber foods (whole grains, vegetables, and fruits) and reduce consumption of high-sugar and high-fat foods. Dietitians provided weekly dietary counseling sessions, and adherence was reinforced through regular follow-up and dietary recall records.

### 2.3. Data collection

All data were collected by trained clinical staff using standardized measurement procedures at baseline and after 12 weeks of intervention to ensure reliability and consistency.

1. Demographic and general information: Demographic and anthropometric parameters, including age, sex, height, and weight, were recorded. BMI was calculated as weight in kilograms divided by the square of height in meters (kg/m²). Waist and hip circumferences were measured to the nearest 0.1 cm using a standardized nonelastic measuring tape, and the waist-to-hip ratio was derived to assess central adiposity. Blood pressure was measured in the seated position after at least 10 minutes of rest using a calibrated electronic sphygmomanometer, and the mean value of 2 consecutive measurements was used for analysis.
2. Body Composition: Body composition indices, including body fat percentage (BF%), lean body mass, and visceral fat area, were assessed using bioelectrical impedance analysis (InBody 770, Biospace Co., Seoul, Korea). All measurements were performed in a fasting state, with participants wearing light clothing and no metal accessories to ensure accuracy.
3. Physical function: Physical function was evaluated through a series of physiological performance indicators, including resting heart rate, step test performance, and vital capacity. Resting heart rate was measured after a 5-minute seated rest using a pulse monitor. The step test was performed following the Harvard step test protocol, and recovery heart rate was recorded to estimate cardiopulmonary endurance. Vital capacity was measured using a portable spirometer (MicroLab 3500, CareFusion, UK), and the best value of 3 consecutive trials was recorded.
4. Physical fitness: Physical fitness was assessed using standardized field tests commonly applied in adolescent health evaluation. The following 5 items were included: standing long jump to evaluate lower limb explosive strength; sit-ups to assess abdominal muscle endurance; sit-and-reach test to measure flexibility; 50-m sprint to assess speed and reaction ability; and 50-m × 8 shuttle run to evaluate aerobic endurance and agility. Each test was performed under the supervision of trained instructors, and the best performance from 2 attempts was recorded for analysis.

All collected data were verified by 2 independent researchers and entered into a secured electronic database. Participants were identified using anonymized study codes to ensure confidentiality and data integrity.

### 2.4. Statistical analysis

All statistical analyses were performed using Statistical Package for the Social Sciences version 27.0 (IBM Corp., Armonk). Continuous variables were assessed for normality using the Shapiro–Wilk test. Variables that met assumptions of normal distribution were expressed as mean ± standard deviation. Categorical variables were summarized as counts and percentages (n, %). Between-group comparisons at baseline (control group vs observation group) were conducted using independent-samples *t* tests for continuous variables and the chi-square test for categorical variables. These analyses were used to confirm comparability of demographic characteristics, anthropometric indicators, body composition indices, physical function, and physical fitness before intervention. Intervention effects over 12 weeks were evaluated in 2 ways. First, post-intervention values at 12 weeks were compared between the 2 groups using independent-samples *t* tests. Second, within-subject change scores were calculated as the difference between the post-intervention value and the baseline value for each participant (Δ = 12-week value − baseline value). These change scores were then compared between groups using independent-samples *t* tests to assess the magnitude of improvement attributable to the intervention. For descriptive purposes, within-group changes from baseline to 12 weeks were also examined using paired *t* tests. For time-dependent performance measures (e.g., 50-m sprint time and 50 m × 8 shuttle run time), lower values were interpreted as better performance. For these variables, negative Δ values indicate improvement. Two-sided p values were reported. A *P* value < .05 was considered statistically significant.

## 3. Results

### 3.1. Baseline characteristics

A total of 129 adolescents with obesity were included in the analysis, with 61 patients in the control group (aerobic exercise only) and 68 patients in the observation group (aerobic exercise plus nutritional intervention). Baseline demographic, anthropometric, hemodynamic, and body composition indicators are summarized in Table 1. The mean age was 15.2 ± 1.6 years in the control group and 15.1 ± 1.5 years in the observation group, with no significant difference between groups (*t* = 0.366, *P* = .715). The proportion of male participants was 59.0% (36/61) in the control group and 63.2% (43/68) in the observation group, and sex distribution did not differ significantly (χ² = 0.241, *P* = .623). BMI (30.8 ± 3.4 kg/m² vs 31.1 ± 3.6 kg/m², *t* = −0.485, *P* = .628) and waist circumference (98.7 ± 8.5 cm vs 99.4 ± 9.1 cm, *t* = −0.450, *P* = .654) were comparable between groups at baseline. Resting systolic blood pressure (128.4 ± 11.2 mm Hg vs 129.1 ± 10.8 mm Hg, *t* = −0.361, *P* = .719) and diastolic blood pressure (79.6 ± 8.4 mm Hg vs 80.1 ± 8.0 mm Hg, *t* = −0.346, *P* = .730) showed no statistically significant differences. BF% measured by bioelectrical impedance analysis was also similar between the control and observation groups (35.2% ± 5.1% vs 35.7% ± 5.3%, *t* = −0.545, *P* = .587). No baseline variable differed significantly between groups (all *P* > .05), indicating the 2 groups were comparable before the intervention.

**Table 1:** Baseline characteristics of adolescents with obesity in the control group and observation group.

Variable	Control group (n = 61)	Observation group (n = 68)	Test statistic	*P*-value
Age (yr)	15.2 ± 1.6	15.1 ± 1.5	*t* = 0.366	.715
Male sex, n (%)	36 (59.0)	43 (63.2)	χ² = 0.241	.623
Body mass index (kg/m²)	30.8 ± 3.4	31.1 ± 3.6	*t* = −0.485	.628
Waist circumference (cm)	98.7 ± 8.5	99.4 ± 9.1	*t* = −0.450	.654
Systolic blood pressure (mm Hg)	128.4 ± 11.2	129.1 ± 10.8	*t* = −0.361	.719
Diastolic blood pressure (mm Hg)	79.6 ± 8.4	80.1 ± 8.0	*t* = −0.346	.730
Body fat percentage (%)	35.2 ± 5.1	35.7 ± 5.3	*t* = −0.545	.587

### 3.2. Body composition and anthropometric outcomes

BMI, waist circumference, and body composition parameters were evaluated at baseline and after 12 weeks in both groups (Table 2). At baseline, there were no significant differences between the control group (aerobic exercise only) and the observation group (aerobic exercise plus nutritional intervention) in BMI, waist circumference, BF%, lean body mass, or visceral fat area (all *P* > .05). After 12 weeks, the observation group showed greater improvement in obesity-related measures. Compared with the control group, the observation group had a lower BMI (28.9 ± 3.2 kg/m² vs 30.2 ± 3.3 kg/m²; *P* = .024), a smaller waist circumference (90.5 ± 7.9 cm vs 95.8 ± 8.1 cm; *P* < .001), a lower BF% (31.5% ± 4.8% vs 33.9% ± 5.0%; *P* = .006), and a smaller visceral fat area (103.5 ± 17.4 cm² vs 111.7 ± 18.2 cm²; *P* = .009). Lean body mass at 12 weeks was slightly higher in the observation group (51.9 ± 7.4 kg vs 50.0 ± 7.1 kg), but this difference was not statistically significant (*P* = .137). The magnitude of change over the 12-week period favored the observation group. Reductions in BMI, waist circumference, BF%, and visceral fat area were greater in the observation group than in the control group, and the increase in lean body mass was also greater in the observation group (all *P* < .001).

**Table 2:** Comparison of BMI, waist circumference, and body composition parameters between the control group and the observation group.

Parameter	Time point	Control group (n = 61)	Observation group (n = 68)	Test statistic	*P*-value
Body mass index (kg/m²)	Baseline	30.8 ± 3.4	31.1 ± 3.6	*t* = −0.487	.627
	12 wk	30.2 ± 3.3	28.9 ± 3.2	*t* = 2.266	.024
	Change (Δ)	−0.6 ± 0.7	−2.2 ± 0.9	*t* = 11.329	<.001
Waist circumference (cm)	Baseline	98.7 ± 8.5	99.4 ± 9.1	*t* = −0.452	.652
	12 wk	95.8 ± 8.1	90.5 ± 7.9	*t* = 3.754	<.001
	Change (Δ)	−2.9 ± 3.4	−8.9 ± 4.1	*t* = 9.079	<.001
Body fat percentage (%)	Baseline	35.2 ± 5.1	35.7 ± 5.3	*t* = −0.546	.585
	12 wk	33.9 ± 5.0	31.5 ± 4.8	*t* = 2.774	.006
	Change (Δ)	−1.3 ± 2.1	−4.2 ± 2.4	*t* = 7.319	<.001
Lean body mass (kg)	Baseline	49.8 ± 7.2	50.1 ± 7.5	*t* = −0.232	.817
	12 wk	50.0 ± 7.1	51.9 ± 7.4	*t* = −1.487	.137
	Change (Δ)	+0.2 ± 1.5	+1.8 ± 1.8	*t* = −5.503	<.001
Visceral fat area (cm²)	Baseline	115.4 ± 18.6	116.2 ± 19.1	*t* = −0.241	.810
	12 wk	111.7 ± 18.2	103.5 ± 17.4	*t* = 2.608	.009
	Change (Δ)	−3.7 ± 5.5	−12.7 ± 6.0	*t* = 8.888	<.001

BMI = body mass index.

### 3.3. Physical function outcomes

Physical function was assessed using resting heart rate, step test performance, and vital capacity at baseline and after 12 weeks (Table 3). At baseline, there were no significant differences between the control group (aerobic exercise only, n = 61) and the observation group (aerobic exercise plus nutritional intervention, n = 68) in resting heart rate (84.2 ± 8.1 bpm vs 83.7 ± 8.4 bpm; *t* = 0.344, *P* = .731), step test index (58.3 ± 6.5 vs 58.1 ± 6.7; *t* = 0.172, *P* = .864), or vital capacity (2.58 ± 0.42 L vs 2.61 ± 0.45 L; *t* = −0.392, *P* = .696). After 12 weeks, the observation group showed greater cardiopulmonary improvement. Resting heart rate was lower in the observation group compared with the control group (76.2 ± 7.5 bpm vs 80.5 ± 7.9 bpm; *t* = 3.161, *P* = .002). The observation group also demonstrated better step test performance (68.3 ± 6.9 vs 62.4 ± 6.8; *t* = −4.886, *P* < .001) and higher vital capacity (2.96 ± 0.47 L vs 2.71 ± 0.44 L; *t* = −3.120, *P* = .002). Analysis of change over time showed larger improvements in the observation group. The reduction in resting heart rate was greater in the observation group (−7.5 ± 3.8 bpm) than in the control group (−3.7 ± 3.5 bpm; *t* = 5.912, *P* < .001). The increase in step test index was greater in the observation group (+10.2 ± 3.0) than in the control group (+4.1 ± 2.9; *t* = −11.735, *P* < .001). Vital capacity increased more in the observation group (+0.35 ± 0.20 L) than in the control group (+0.13 ± 0.18 L; *t* = −6.576, *P* < .001).

**Table 3:** Comparison of physical function between the control group and the observation group.

Parameter	Time point	Control group (n = 61)	Observation group (n = 68)	Test statistic	*P*-value
Resting heart rate (bpm)	Baseline	84.2 ± 8.1	83.7 ± 8.4	*t* = 0.344	.731
	12 wk	80.5 ± 7.9	76.2 ± 7.5	*t* = 3.161	.002
	Change (Δ)	−3.7 ± 3.5	−7.5 ± 3.8	*t* = 5.912	<.001
Step test index (score)	Baseline	58.3 ± 6.5	58.1 ± 6.7	*t* = 0.172	.864
	12 wk	62.4 ± 6.8	68.3 ± 6.9	*t* = −4.886	<.001
	Change (Δ)	+4.1 ± 2.9	+10.2 ± 3.0	*t* = −11.735	<.001
Vital capacity (L)	Baseline	2.58 ± 0.42	2.61 ± 0.45	*t* = −0.392	.696
	12 wk	2.71 ± 0.44	2.96 ± 0.47	*t* = −3.120	.002
	Change (Δ)	+0.13 ± 0.18	+0.35 ± 0.20	*t* = −6.576	<.001

### 3.4. Physical fitness outcomes

Physical fitness was evaluated using standardized field performance tests, including lower limb explosive strength (standing long jump), abdominal muscle endurance (sit-ups), flexibility (sit-and-reach), speed (50-m sprint), and aerobic endurance/agility (50 m × 8 shuttle run). Results are summarized in Table 4. At baseline, there were no significant differences between the control group (aerobic exercise only, n = 61) and the observation group (aerobic exercise plus nutritional intervention, n = 68) in standing long jump distance, number of sit-ups, sit-and-reach distance, 50-m sprint time, or shuttle run time (all *P* > .05), indicating comparable physical fitness status before intervention. After 12 weeks, the observation group demonstrated superior performance in multiple domains. Compared with the control group, the observation group achieved a longer standing long jump distance (182.0 ± 18.0 cm vs 171.0 ± 19.0 cm; *t* = −3.366, *P* = .001), completed more sit-ups in 30 seconds (31.0 ± 5.0 vs 27.0 ± 5.0; *t* = −4.536, *P* < .001), and showed greater flexibility (sit-and-reach 10.6 ± 4.5 cm vs 8.4 ± 4.3 cm; *t* = −2.838, *P* = .005). The observation group also recorded faster 50-m sprint times (8.2 ± 0.6 s vs 8.6 ± 0.7 s; *t* = 3.465, *P* < .001) and faster 50 m × 8 shuttle run times (77.4 ± 6.2 s vs 81.1 ± 6.5 s; *t* = 3.299, *P* = .001), indicating better speed, agility, and aerobic endurance.

**Table 4:** Comparison of physical fitness performance between the control group and the observation group.

Parameter	Time point	Control group (n = 61)	Observation group (n = 68)	Test statistic	*P*-value
Standing long jump (cm)	Baseline	165.0 ± 18.0	166.0 ± 18.0	*t* = −0.315	.753
	12 wk	171.0 ± 19.0	182.0 ± 18.0	*t* = −3.366	.001
	Change (Δ cm)	+6.0 ± 6.0	+17.0 ± 7.0	*t* = −9.608	<.001
Sit-ups (reps/30 s)	Baseline	24.0 ± 5.0	24.0 ± 5.0	*t* = 0.000	1.000
	12 wk	27.0 ± 5.0	31.0 ± 5.0	*t* = −4.536	<.001
	Change (Δ reps)	+3.0 ± 2.0	+7.0 ± 2.0	*t* = −11.341	<.001
Sit-and-reach (cm)	Baseline	7.2 ± 4.1	7.0 ± 4.2	*t* = 0.273	.785
	12 wk	8.4 ± 4.3	10.6 ± 4.5	*t* = −2.838	.005
	Change (Δ cm)	+1.2 ± 1.5	+3.6 ± 1.6	*t* = −8.791	<.001
50-m sprint (s)	Baseline	8.9 ± 0.7	8.8 ± 0.7	*t* = 0.810	.419
	12 wk	8.6 ± 0.7	8.2 ± 0.6	*t* = 3.465	<.001
	Change (Δ s)	−0.3 ± 0.3	−0.6 ± 0.3	*t* = 5.671	<.001
50 m × 8 shuttle run (s)	Baseline	83.5 ± 6.8	83.2 ± 6.7	*t* = 0.252	.802
	12 wk	81.1 ± 6.5	77.4 ± 6.2	*t* = 3.299	.001
	Change (Δ s)	−2.4 ± 2.0	−5.8 ± 2.1	*t* = 9.414	<.001

## 4. Discussion

This study evaluated the effects of aerobic training alone versus aerobic training combined with structured nutritional intervention on anthropometric parameters, body composition, cardiopulmonary function, and physical fitness in adolescents with obesity. The 2 groups were comparable at baseline with respect to age, sex distribution, blood pressure, BMI, waist circumference, and BF%, which supports the internal comparability of the cohorts. After 12 weeks, adolescents who received combined aerobic exercise and nutritional intervention demonstrated greater reductions in BMI, waist circumference, total BF%, and visceral fat area than those who received aerobic exercise alone. In parallel, this group also showed greater improvements in lean body mass, resting heart rate, step test performance, vital capacity, standing long jump performance, sit-up performance, flexibility, sprint speed, and shuttle run time. These findings suggest that adding nutritional intervention to aerobic exercise is associated with more favorable changes in both adiposity-related indicators and functional capacity in obese adolescents.

The reduction in BMI and waist circumference in the observation group is clinically relevant. BMI is an established indicator of total adiposity, while waist circumference is a marker of central adiposity that correlates with visceral fat accumulation and cardiometabolic risk in youth. Central obesity in particular is linked to insulin resistance, hypertension, dyslipidemia, and later cardiovascular morbidity. Reductions in waist circumference of the magnitude observed in this study (approximately 9 cm over 12 weeks in the combined-intervention group) indicate not only weight control but also redistribution or loss of metabolically active visceral fat. These data align with the observation that visceral fat area decreased more in the combined-intervention group than in the aerobic-only group. Visceral adiposity is considered a high-risk fat depot in adolescents and is associated with early vascular and metabolic alterations. Sustained reduction in visceral fat in adolescence is thought to attenuate the trajectory toward adult metabolic syndrome.^[11,12]^

The improvement in lean body mass observed in the group receiving both aerobic exercise and nutritional counseling is also notable. Weight loss interventions in adolescents often lead to parallel reductions in fat mass and lean mass, and preservation or gain of lean mass is considered desirable because skeletal muscle contributes to resting energy expenditure, glucose disposal, and physical performance. Prior pediatric trials have reported that structured exercise can stabilize or increase fat-free mass despite overall weight reduction, although such effects are not universal and may depend on training mode and dietary adequacy.^[13,14]^ In the current analysis, the combined-intervention group demonstrated a greater increase in lean mass than the aerobic-only group, suggesting that coordinated nutrition may have supported protein intake and recovery, thereby facilitating favorable body recomposition rather than simple weight loss.

Beyond body composition, the combined intervention produced superior improvements in cardiopulmonary indicators. Resting heart rate decreased to a greater extent in the observation group, and the step test index and vital capacity improved more markedly. Resting heart rate in adolescents reflects autonomic regulation and cardiorespiratory fitness. The step test is an index of submaximal cardiovascular endurance, whereas vital capacity reflects ventilatory function and thoracic–abdominal mechanics. The observed pattern indicates that integrating nutritional intervention with aerobic exercise may amplify gains in aerobic conditioning and respiratory function compared with exercise alone, possibly through improved training tolerance, recovery, and body mass-supported mechanical efficiency. Similar exercise-based interventions in children and adolescents with overweight or obesity have been shown to improve cardiorespiratory fitness, reduce cardiometabolic risk scores, and decrease visceral adipose tissue.^[15]^

Physical fitness performance, including lower limb explosive strength (standing long jump), core muscular endurance (sit-ups), flexibility (sit-and-reach), sprint speed, and shuttle run performance, also improved more in the combined-intervention group than in the aerobic-only group. These outcomes extend the interpretation of the intervention beyond weight-centric endpoints. Enhanced sprint performance and shuttle run times suggest improved neuromuscular coordination, anaerobic capacity, and agility, in addition to aerobic endurance. The concurrent gains in sit-ups and sit-and-reach indicate improvements in trunk endurance and flexibility, which are important for injury prevention, posture, and functional mobility in daily activities. Notably, in many pediatric weight management programs, physical function and movement quality are not routinely reported despite their relevance to long-term exercise adherence and quality of life.^[16,17]^ The present findings support the view that benefits of combined lifestyle intervention in obese adolescents are multidimensional and include not only fat loss but also functional physical capacity.

The current findings are consistent with, and extend, recent evidence that structured exercise programs can induce measurable improvements in adiposity, visceral fat distribution, and cardiorespiratory fitness in youth with obesity. A randomized clinical trial in children with overweight and obesity reported that a 20-week combined aerobic and resistance training program significantly reduced BMI, total fat mass, and visceral adipose tissue while improving cardiorespiratory fitness, and also lowered a clustered cardiometabolic risk score relative to controls. That trial emphasized that exercise exerts synergistic effects across metabolic domains, supporting the concept of exercise as a “multicomponent intervention” for cardiometabolic health. The present study observed similar trends in adolescents over a 12-week period, including reductions in BMI and visceral fat area and improvements in cardiopulmonary indicators, suggesting that clinically relevant adaptations can emerge within a relatively short timeframe.

Recent meta-analytic evidence further supports the role of aerobic exercise in modifying key obesity-related indicators in pediatric populations. A 2025 systematic review of randomized controlled trials in overweight and obese children and adolescents concluded that aerobic training alone significantly reduces BMI, waist circumference, total BF%, and visceral adipose tissue.^[18]^ These findings align with the improvements observed in the aerobic-only control group in the present analysis, in whom BMI, waist circumference, and resting heart rate also improved over 12 weeks. However, the magnitude of change was consistently greater in the group that received combined aerobic exercise and nutritional intervention, indicating that exercise alone is beneficial but may be suboptimal when compared with integrated strategies. Nutritional intervention appears to be a key intensifier of response. A 12-week supervised program that integrated structured physical training with unified dietary guidance in obese adolescents produced larger reductions in body mass, BMI, and BF% than exercise training without such dietary standardization.^[19]^ This pattern mirrors the present results, in which the group receiving both aerobic training and nutrition support exhibited greater declines in BMI, waist circumference, and visceral fat area, and greater gains in lean mass, than the aerobic-only group. These converging data suggest that alignment between energy expenditure (exercise) and controlled energy/intake quality (diet) is important for optimizing both fat loss and muscle preservation in adolescents with obesity.

Central adiposity is particularly relevant. A 2025 systematic review and meta-analysis of randomized trials on pediatric central obesity reported that interventions combining dietary modification and physical activity were associated with significant reductions in waist circumference, whereas physical activity alone did not consistently achieve the same magnitude of central fat reduction.^[20]^ The present study demonstrated a significantly smaller waist circumference and visceral fat area at 12 weeks in the combined-intervention group compared with the aerobic-only group, consistent with the interpretation that multimodal approaches are more effective in targeting abdominal fat depots in adolescents. In terms of functional outcomes, recent pediatric trials have shown that structured exercise improves cardiorespiratory fitness and certain performance metrics, but effects on neuromuscular function and speed-agility components are variable and may depend on the training mode. The current findings indicate that coupling aerobic training with nutritional intervention was associated with superior improvements not only in endurance-oriented metrics (shuttle run, step test index) but also in explosive strength and speed (standing long jump distance, 50-m sprint time). This suggests that body mass reduction itself may facilitate better mechanical efficiency and movement economy, thereby translating into faster sprint times and longer jump distances. Finally, accumulating evidence indicates that lifestyle interventions that incorporate physical activity can reduce cardiovascular disease risk markers and improve metabolic regulation in overweight and obese youth, potentially through enhanced insulin signaling, improved lipid handling, and reductions in blood pressure and central adiposity. The concomitant reduction in resting heart rate and improvement in ventilatory capacity observed in the present study are consistent with these cardioprotective trajectories.

The findings support implementation of multidisciplinary, clinic-based programs for obese adolescents that integrate aerobic exercise with individualized nutritional guidance rather than prescribing exercise alone. Such programs may achieve clinically meaningful reductions in BMI and visceral adiposity within 3 months, while simultaneously improving cardiorespiratory fitness, muscular endurance, flexibility, and speed-agility performance. These multidimensional gains are relevant for reducing early cardiometabolic risk, enhancing functional capacity required for daily activity and sport participation, and improving the likelihood of long-term behavioral adherence during adolescence, a developmental period when obesity tends to persist into adulthood. This study has several strengths. First, it directly compared aerobic training alone to aerobic training combined with structured nutritional intervention within the same clinical setting, allowing evaluation of the incremental effect of dietary management. Second, it assessed a broad range of endpoints beyond body weight, including visceral fat area, lean body mass, resting heart rate, ventilatory capacity, and standardized field fitness tests. This multidimensional assessment captures both metabolic risk and physical functioning, which is important for adolescent health. Third, the intervention period of 12 weeks is feasible for outpatient or school-affiliated programs, which increases translational relevance.

Several limitations should be acknowledged. The study used a retrospective design rather than random allocation, which introduces the possibility of selection bias and unmeasured confounding. Although baseline characteristics did not differ significantly between groups, residual confounding cannot be excluded. Nutritional intake in the aerobic-only group was not actively standardized, so differences in outcomes could partly reflect adherence or motivation rather than the intervention itself. Follow-up was limited to 12 weeks, and long-term weight maintenance, metabolic adaptation, and behavioral sustainability were not assessed. The study did not directly measure biochemical markers such as fasting glucose, insulin resistance indices, lipid profiles, or inflammatory markers, which limits inference about cardiometabolic risk modification. Finally, the analysis was conducted in a single center, which may limit generalizability to other demographic or sociocultural settings.

## 5. Conclusions

In this study of obese adolescents, a 12-week program combining aerobic exercise with nutritional intervention produced significantly greater improvements in BMI, waist circumference, total and visceral body fat, and lean body mass than aerobic exercise alone. The combined approach also enhanced cardiopulmonary function and multiple aspects of physical fitness, including strength, endurance, flexibility, and agility. These findings indicate that integrated exercise and dietary strategies are more effective than single-component interventions for optimizing body composition and physical performance in adolescent obesity management.

## Author contributions

**Conceptualization:** Yahui Chang, Bo Zhou, Yalan Zhang, Ziyang He, Haoxuan Song, Yang Wang, Meng Su.

**Data curation:** Yahui Chang, Bo Zhou, Yalan Zhang, Ziyang He, Haoxuan Song, Yang Wang, Shurui Li, Meng Su.

**Formal analysis:** Yahui Chang, Bo Zhou, Yalan Zhang, Ziyang He, Haoxuan Song, Yang Wang, Shurui Li, Meng Su.

**Funding acquisition:** Yahui Chang, Bo Zhou, Ziyang He, Shurui Li, Meng Su.

**Investigation:** Meng Su.

**Writing – original draft:** Yahui Chang, Bo Zhou, Ziyang He, Shurui Li, Meng Su.

**Writing – review & editing:** Yahui Chang, Bo Zhou, Ziyang He, Shurui Li, Meng Su.
